# Antimicrobial activity of *Plectranthus barbatus *(Lamiacea)

**DOI:** 10.1186/1753-6561-8-S4-P264

**Published:** 2014-10-01

**Authors:** Regina Célia Sales Santos Veríssimo, Thais Honório Lins, Maria Lysete de Assis Bastos, Patrícia de Albuquerque Sarmento, Valter Alvino, Maria Gabriella Silva Araujo, Andressa Letícia Lopes Silva, João Xavier Araújo-Júnior

**Affiliations:** 1 Federal University of Alagoas, Maceió, AL, Brazil

## Introduction

The medicinal potential of plant is due to the presence of active principles, capable of producing several pharmacological effects such as analgesic, antiseptic agents, diuretics, expectorants, tranquilizers, digestive healing, emollients, anti-diarrhoeal, among others [1]. The deep cultural roots of the Brazilian population facilitated the permanence of the use of herbal medicine to the present day acknowledging its effectiveness and legitimacy. [2] A strong scientific interest in herbal medicine has grown in recent years, which has led to the development of several studies that were based on popular practices with the use of plants for various therapeutic purposes [3]. One of medicinal plants, *Plectranthus barbatus*, is traditionally used as anti-inflammatory and antifungal agents, and has been recognized for its effects against the bacterias that cause dental caries such as *Streptococcus mutans *and *Streptococcus sobrinus *[4].

## Objective

To evaluate the antimicrobial potential of leaf extracts from *Plectranthus barbatus *front of species which often causing infection of wounds.

## Methodology

The extracts were prepared by maceration in 96% ethanol. The crude ethanol extract was tested against microorganisms: *Staphylococcusaureus, Staphylococcusepidermidis, Pseudomonasaeruginosa, Enterobacteraerogenes, Salmonellaenterica, Streptococcuspneumoniae, Acinetobactercalcoaceticus, EscherichiacoliandCandidaalbicans *by agar perforation methods. Subsequently, it was determined the Minimum Inhibitory Concentration (MIC) by the broth micro dilution method using the culture media Brain Heart Infusion (BHI) broth in 96-well plates. The solubilization of the samples was performed with saline solution 0.9% and Tween 80. The positive control used was Ceftriaxone. It was used as a developer 2,3,5-triphenyl tetrazolium chloride (0.5 mg / ml). All experiments were performed in triplicate. Statistical analysis was performed using Kruskal-Wallis test. Data were considered significant when p <0,05.

## Results and conclusion

The extract showed zone of inhibition average of 14,33 (± 0,47) against *Staphylococcus aureus *is considered active according to the methodology AYRES [5]. The MIC had favourable outcomes for *Staphylococcus aureus *(3,12 mg/ml) *Staphylococcus epidermidis *(6,25mg/ml), *Streptococcus pneumoniae *(6,25 mg/ml) and *Escherichia coli (6,25 mg/ml)*. The results allowed to conclude that *Plectranthus barbatus *possesses the ability to inhibit pathogenic bacteria, proving that possesses antimicrobial activity that are prospects for obtaining natural antibiotics. The healing properties, their cytotoxic potential, as well as the development of bioproducts, specifically for infected wound, are underway.
